# PcLRR-RK3, an LRR receptor kinase is required for growth and *in-planta* infection processes in *Phytophthora capsici*

**DOI:** 10.1080/21501203.2024.2305720

**Published:** 2024-01-31

**Authors:** Asma Safdar, Feng He, Danyu Shen, Muhammad Imran Hamid, Sajid Aleem Khan, Hafiz Abdul Samad Tahir, Daolong Dou

**Affiliations:** a Department of Plant Pathology, College of Plant Protection, Nanjing Agricultural University, Nanjing, China; b Department of Plant Pathology, College of Agriculture, University of Sargodha, Sargodha, Pakistan; c College of Life Sciences, Anhui Normal University, Wuhu, China; d Department of Botany and Plant Sciences, University of California, Riverside, CA, USA; e Department of Plant Pathology, University of Agriculture, Faisalabad, Pakistan

**Keywords:** LRR-RLKs, vegetative growth, pathogenicity, *Phytophthora capsici*

## Abstract

Receptor protein kinases (RPKs) critically provide the basic infrastructure to sense, perceive, and conduct the signalling events at the cell surface of organisms. The importance of LRR-RLKs has been well studied in plants, but much less information has been reported in oomycetes. In this work, we have silenced the PcLRR-RK3 and characterised its functional importance in *Phytophthora capsici*. PcLRR-RK3 was predicted to encode signal peptides, leucine-rich repeats, transmembrane, and kinase domains. PcLRR-RK3-silenced transformants showed impaired colony growth, decreased deformed sporangia, and reduced zoospores count. The mycelium of silenced transformants did not penetrate within the host tissues and showed defects in the pathogenicity of *P. capsici*. Interestingly, gene silencing also weakens the ability of zoospores germination and penetration into host tissues and fails to produce necrotic lesions. Furthermore, PcLRR-RK3 localisation was found to be the plasma membrane of the cell. Altogether, our results revealed that PcLRR-RK3 was required for the regulation of vegetative growth, zoospores penetration, and establishment into host leaf tissues.

## 1. Introduction

Leucine-rich repeat receptor-like kinases (LRR-RLKs) have been categorised as the largest subfamily of transmembrane receptor-like kinases with more than 200 members in *Arabidopsis* and more than 300 members in rice, regulating various host defence responses (Torii 2004; Wu et al. 2016). The kinase receptors are involved in signal transduction via the cell surface to regulate the various cellular processes in living organisms. LRR-RLKs are involved in a wide range of biological signalling processes and host defence responses by providing the backbone to mediate the interaction with other polypeptides (Stø et al. 2015) and directly participating in various developmental functions (Torii 2004; Geng et al. 2021). The existence of LRR-RLKs has been reported in many life forms, but phylogenetic analysis indicated that LRR-RLKs have an ancient origin with more recent expansion in plant lineages (Han and Bleecker 2003). Another phylogenetic analysis showed that LRR-RLKs were completely absent in fungi but not in oomycetes species which suggested that oomycetes must have some distinct strategies for perceiving and responding to environmental signals (Soanes et al. 2010). Later, Diévart et al. (2011) showed that LRR-RLKs are present in all oomycetes species with an evolutionary history consistent with plants. LRR-RLKs subfamily in oomycetes consists of the developed, expanded, and functionally diversified subfamily of LRR-RLKs which was structurally but not phylogenetically similar to plants.

Oomycetes comprise a diverse group of organisms with diverse lifestyles that can live as saprophytes or parasites and cause diseases in plants, vertebrates, fish, microbes, and insects (Govers and Gijzen 2006). Oomycetes are morphologically similar to filamentous fungi and have common infection mechanisms (Latijnhouwers et al. 2003). However, phylogenetically, oomycetes together with brown algae and diatoms are placed under Straminopiles, a group more closely related to algae and plants than fungi (Weerakoon et al. 1998; Baldauf et al. 2000; Förster et al. 2000). Unlike fungi, oomycetes are usually diploid with higher ploidy levels within species. Over the past few decades, there has been a significant expansion in the number of identified species within this genus (Martin 2000; Reeser et al. 2007; Bertier et al. 2013; Yang et al. 2014).

Oomycetes have biotrophic to necrotrophic lifestyles with narrow to broader host ranges and can cause damage to a large number of crops worldwide. This diverse lifestyle of oomycetes explains the evolution of the gene family in Chromista, particularly oomycetes by gain and loss of genes (Martens et al. 2008; Martin et al. 2014). Oomycete species possessed significant large and diverse sets of expanded gene families (Tyler et al. 2006; Levesque et al. 2010; Seidl et al. 2011). The gene families have been expanded because of the gene gain, duplications and losses, abundance, and presence/absence pattern between the species (Seidl et al. 2012). LRR-RLKs gene families are also distributed in the oomycetes genome and expressed differentially during the infection process (Diévart et al. 2011). A total of ten LRR-RLKs genes are identified in the *P. capsici* genome (Safdar et al. 2017). *Phytophthora capsici* Leonian, one of the most notorious oomycetes species, is a soil-borne pathogen that has a wide host range in many *Solanaceae* and *Cucurbitaceae* families (Granke et al. 2012) and caused significant crop damage worldwide (Kroon et al. 2011).

Taking into consideration of importance and functional diversity of LRR-RLKs, we investigated their importance in oomycetes. In this study, we have selected the PcLRR-RK3 for functional characterisation in the development and pathogenicity of *P. capsici.*

## 2. Material and methods

### 2.1. Protein sequence selection and identification

DOE JGI database (http://genome.jgi.doe.gov/) was used to download the *P. capsici* (v1.0) genomic protein sequence (Lamour et al. 2012). We used the Pfam database (http://pfam.xfam.org/) to check the hidden Markov models of eight known LRR domains (accession numbers: PF00560, PF07723, PF07725, PF12799, PF13306, PF13516, PF13855, and PF14580) using HMMER tool with *E*-value = 1e^−5^ as the cut-off. The transmembrane domain was predicted in each protein sequence using the TMHMM server (Krogh et al. 2001). SignalP 3.0 (Bendtsen et al. 2004) was used to detect signal peptides with HMM score >0.9 as the cut-off.

### 2.2. *Maintenance of cultures of* P. capsici *and plants*

The fresh *P. capsici* (LT263) culture was taken and maintained in the laboratory on 10% V8 agar medium at 25 °C in the dark. All pathogenicity assays were performed on tobacco and *Arabidopsis* plants kept in the greenhouse at 25 °C (Mafurah et al. 2015).

### 2.3. *Construction of plasmid and gene transformation into* P. capsici *culture*

The DNA fragment (598 bp) of *PcLRR-RK3* (Gene ID: Pc525500) was amplified using LA Taq polymerase (TaKaRa) and primers Pc525500SmaIF (5ʹ-CCCGGGacaacaATGCAAGCGTGGTTGACTTTGG-3ʹ) and Pc525500KpnIR (5ʹ-GCACCGGGGTACCTTAAAGGCTTCGTCTTGGAAGTT-3ʹ). The PCR product and expression vector pHam34 were digested with the restriction enzymes *Sma*I and *Kpn*I and ligated for their cloning. The DNA sequencing was performed for verification of insertions (Dong et al. 2015).

To generate the GFP: PcLRR-RK3 for gene overexpression, we substituted the *PcLRR-RK3* gene with PsAtg8 in GFP-PsAtg8 kindly provided by Chen et al. (2017). The GFP-PsAtg8 contained AcGFP1 gene (AB255038.1) and PsAtg8gene. The full-length PcLRR-RK3 with its signal peptide was amplified using the cDNA of *P. capsici* LT263 as a template with LA *Taq* polymerase (TaKaRa). The primers used were 500-23-SmaI-F (5ʹ-ATACCCGGGATGCAAGCGTGGTTGACTTTGGC-3ʹ) and 500-23-XbaI-R (5ʹ-GCATCTAGACAGCTTCATGGCGCCATTCTTAAAAG-3ʹ). The candidate *PcLRR-RK3* gene was inserted into GFP-PsAtg8 at 3′ terminal of *AceGFP* gene with restriction sites of *SmaI* and *XbaI*. The resulting expression vector GFP: PcLRR-RK3 contained the candidate gene and a C-terminal fused GFP.

The polyethylene glycol (PEG)-mediated protoplast transformation method was used for gene transformation (McLeod et al. 2008). The sprouted mycelium was picked and multiplied separately in agar plates containing 10% V8 medium with 30 μg/mL G418. The transcript level of the gene was quantified by qRT-PCR in the mycelium of putative transformants.

### 2.4. RNA extraction and qRT-PCR assay

The *P. capsici* (LT263) and transformants, cultured in 10% V8 media for 3 days, were collected directly into liquid nitrogen and stored at −70 °C for DNA or RNA extraction. Genomic DNA extraction was performed by the CTAB method, and RNA extraction was performed according to the manufacturer’s protocols (RNAsimple Total RNA kit, Tiangen) (Wang et al. 2016). Agarose gel electrophoresis and a spectrophotometer (Nanodrop ND-1000) were used to assure the RNA quality.

The total RNA was converted into cDNA using a kit (PrimeScript reagent Kit, TaKaRa). The synthesised cDNA was diluted 1:2 with ddH_2_O and used for qRT-PCR analysis. SYBR green qRT-PCR was performed using SYBR Green Master Mix (Vazyme) on an ABI PRISM 7500 Real-Time PCR System (Applied Biosystems). The 20 µL mixture, containing 40 ng of cDNA, 0.2 mmol/L gene-specific primer of PcLRR-RK3, 10 µL of SYBR Green Master Mix (Vazyme), 0.4 µL of ROX Reference Dye 2 and 6.8 µL of dH_2_O, was run to check the transcription rate of PcLRR-RK3 under reaction condition adjusted as described previously by Wang et al. (2016). The transcript level of *PcLRR-RK3* in raw data was normalised using *P. capsici* actin gene (Pc132086) as a reference gene. The primers used were Pc500RTF (5ʹ-GTCTTCCAACCCGCAGAAAG-3ʹ) and Pc500RTR (5ʹ-TGCCAACCTGAATGTCAAGC-3ʹ) to quantify *PcLRR-RK3*.

### 2.5. Phenotypic analysis of PcLRR-RK3-silenced transformants

Mycelia of wild type (LT263) and silenced transformants (T5 and T10) were multiplied on 10% V8 medium without G418 and incubated for 3 days at 25 °C for colony growth assay. Colony diameter was measured once a day and photographs were taken with Canon EOS600D camera.

The number and morphology of zoosporangium were documented by incubating each strain (T5, T10, and WT) in 4 mL of 10% V8 broth at 25 °C for up to 36 h. Then, mycelium of each strain was washed three times with water to induce the sporangium development and incubated again. The data were recorded at 4, 8, 12, and 24 h intervals and DIC images of counted sporangia were taken with Olympus IX-72 microscope.

The zoospores were released by keeping sporangium carrying mycelium plates at 4 °C for 30 min and then transferring at 25 °C for the next 30 min. The 1 μL of zoospores suspension was taken, and the zoospores number was counted at 4, 8, 12, and 24 h intervals. Each experiment contained three biological repeats and was repeated three times. The significance of the data was checked with a one-way analysis of variance (ANOVA) test followed by LSD test.

### 2.6. *Pathogenicity assay on* Nicotiana benthamiana

Virulence assay was performed using mycelial plug inoculation on detached *N. benthamiana* leaves to evaluate the virulence of *PcLRR-RK3-*silenced transformants (T5 and T10) as compared to *P. capsici* LT263 (WT) following Safdar et al. (2017). The lesion formation was examined at 36 and 48 h post-inoculation (hpi) and photographs were taken with a Canon EOS600D camera. The data were analysed statistically with the Student’s *t*-test.

### 2.7. Trypan blue staining, DAB staining, and callose deposition assay

To assess the invasive ability of mycelium of *PcLRR-RK3-*silenced transformants and generation of host defence responses, we performed trypan blue staining, DAB staining, and callose deposition assay. Trypan blue staining was performed as described by Yanagawa et al. (2017). Briefly, *N. benthamiana* leaves were inoculated with hyphal plugs of relevant strains (WT, T5, and T10) and kept at 25 °C. At 24 hpi, the leaves were boiled in a trypan blue stain solution for 5 min. The trypan blue stain solution contained phenol/glycerol/lactic acid/water/ethanol at the ratio of 1:1:1:1:2 by volume. The samples were then decolourised with chloral hydrate solution and observed by light microscopy and photographed.

ROS accumulation was detected by staining the infected leaves with diaminobenzidine (DAB) solution (Zhang et al. 2015). The *N. benthamiana* leaves were inoculated with hyphal plugs of relevant strains (WT, T5, and T10) and kept at 25 °C. After 12 hpi, infected leaves were kept in DAB stain solution for the next 8 h at 25 °C. Then, stained leaves were fixed in 95% ethanol to remove the chlorophyll and photographed. The experiment was repeated at least three times.

Callose deposition assay on *Arabidopsis* plants was performed by inoculating mycelium plugs of relevant strains (WT, T5, and T10) as previously described (Mafurah et al. 2015). The infected leaves were cut out and soaked in (1:1:1:1:8 of phenol:glycerol:lactic acid:water:ethanol) bleaching solution until clearance of chlorophyll. Then, excised leaf tissues were washed with distilled water and dipped in 0.01% aniline blue in 150 mmol/L K_2_HPO_4_ (pH 9.5) and kept for 4 h under dark conditions. The stained leaf tissues were examined under a UV epifluorescence microscope (Olympus IX-72 microscope) by mounting in 50% glycerol. The experiment was repeated three times with an average of ten plants per treatment.

### 2.8. Observation of zoospore germination and infection into host tissues

To observe the germination of zoospores of *PcLRR-RK3-*silenced transformants, we calculated germination cyst (%) by following Chen et al. (2014). The encystment of zoospores was induced by vortexing zoospore suspension (300 μL) for 90 s and incubating at 25 °C after adding an equal volume of 5% (v/v) V8 broth for 2.5 h. The germinated cysts number was counted in 1 μL of suspension and scored germinated if their germ tube length equalled or exceeded the cyst diameter (10 μm). The drops of cyst suspensions were transferred to glass slides and examined under a UV epifluorescence microscope (Olympus IX-72 microscope). Each experiment contained three biological repeats and was repeated three times. The significance of the data was checked with a one-way analysis of variance (ANOVA) test followed by LSD test.

To assess the zoospores’ infectious ability of transformants (T5 and T10), the *N. benthamiana* and *A. thaliana* detached leaves were used for inoculation. The zoospore suspension was prepared as stated above. The detached leaves were inoculated with 10 μL suspension containing 100 zoospores/µL. The experiment was maintained at 25 °C and 80% humidity in the dark. Lesion formation was examined up to 48 h post-inoculation (hpi), and photographs were taken with a Canon EOS600D camera.

For microscopic observation of zoospore germination, penetration, and invasive hyphae expansion, tobacco leaves were inoculated with 10 µL of zoospores suspension of T5 and WT as described (Safdar et al. 2017). The experiment was maintained at 25 °C and 80% humidity in the dark for up to 48 hpi. The data were recorded at 6, 12, 24, and 48 hpi by collecting inoculated leaves at each time point. The collected leaves were excised from the inoculation site, stained with trypan blue, and cleared with chloral hydrate. The leaf tissues were examined and DIC images were taken using a UV epifluorescence microscope (Olympus IX-72 microscope). The experiment was repeated three times with 14 biological repeats.

### 2.9. *Agrobacterium-mediated transient gene expression in* N. benthamiana

To assess gene localisation in *N. benthamiana* leaves, we followed the *Agrobacterium*-mediated transient gene expression analysis as described previously (Rajput et al. 2015). We amplified the full-length gene *PcLRR-RK3* (Pc525500) using the cDNA of *P. capsici* LT263 as a template with LA *Taq* polymerase (TaKaRa). The primers used were Pc525500pBINSmaIF (5ʹ-ATACCCGGGATGCAAGCGTGGTTGACTTTGG-3ʹ) and Pc525500pBINXbaIR (5ʹ-GCTCTAGACATTACAGCTTCATGGCGCCATTCTTA-3ʹ). The PCR product was cloned into vector pBINGFP2 with the restriction enzymes *SmaI* and *XbaI*.

DNA sequencing was conducted to verify the correct insertions. This constructed vector and empty vector were then introduced into *Agrobacterium tumefaciens* strain GV3101 by electroporation method and cultured in Luria – Bertani medium at 28 °C with shaking at 220 r/min for 48 h. The bacterial cultures were washed with 10 mmol/L MgCl_2_ and re-suspended with infiltration buffer (10 mmol/L MgCl_2_, 10 mmol/L MES, pH 5.6, and 150 mmol/L acetosyringone) to an *OD*_600_ of 0.5 and infiltrated into the *N. benthamiana* leaves with a needleless syringe (Rajput et al. 2015). The leaves were harvested after 48 h of incubation for confocal microscopic analysis.

### 2.10. Confocal microscopy

The samples (infiltrated leave tissues and transformed mycelium) were mounted in water on a glass slide under a cover slip for confocal microscopic analysis. The GFP fluorescence was imaged using a Zeiss LSM710 confocal microscope with an excitation wavelength of 488 nm and a 63X oil objective lens. The *N. benthamiana* leaves infiltrated with *Agrobacterium tumefaciens* carrying PcLRR-RK3: GFP was monitored for plant subcellular localisation. The *P. capsici* mycelium transformants carrying PcLRR-RK3: GFP were monitored for *P. capsici* subcellular localisation. The leaves infiltrated with empty vector and *P. capsici* transformed with GFP alone were used as control. The confocal microscopy was performed at least twice for *N. benthamiana* leaves and thrice for *P. capsici* mycelium culture. Images were processed using the Zeiss LSM710 software.

## 3. Results

### 3.1. *PcLRR-RK3 is affecting* P. capsici *growth*

**Figure 1.**
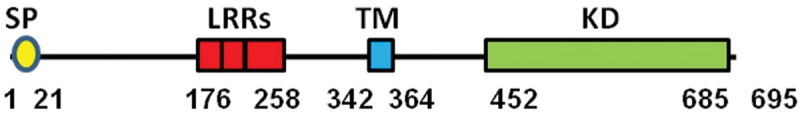
Graphic schematic demonstration of PcLRR-RK3. A protein encoding a 713-amino-acid having signal peptide (SP), three LRRs domains, transmembrane domain (TM), and kinase domain (KD).

A total of ten putative LRR-carrying receptors were identified in *P. capsici* containing LRR, transmembrane, and kinase domains (Safdar et al. 2017). In this study, we selected PcLRR-RK3 (Gene ID: Pc525500) for detailed functional characterisation. Domain searches using indicated database showed that PcLRR-RK3 was a 695 amino acids’ Leucine-rich repeat receptor-kinase protein (LRR-RK) with four domains, an N-terminal signal peptide (1–21 amino acids), three LRR domains (176–258 amino acids), a transmembrane domain (342–264 amino acids) and a kinase domain (452–586 amino acids) (Figure 1).

**Figure 2.**
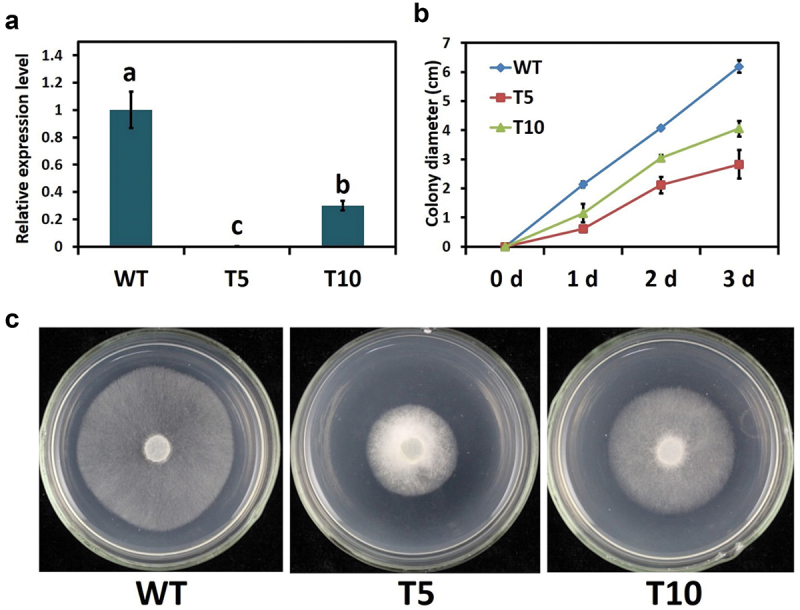
The mycelium growth phenotype in *PcLRR-RK3-*silenced transformants of *Phytophthora capsici*. (a) Relative transcript level of *PcLRR-RK3* in transformants. (b) Growth is reduced in *PcLRR-RK3-*silencedtransformants. (c) Phenotype of colony in *PcLRR-RK3*-silenced transformants compared with wild type (WT).

To explore the possible contribution of PcLRR-RK3 in the biology of *P. capsici*, we used a PEG-mediated transformation strategy and generated PcLRR-RK3-silenced transformants in *P. capsici* strain LT263. We selected two (T5 and T10) out of eleven transformants after antibiotic (G418) screening and qRT-PCR analysis. The T5 and T10 showed about 70% to 100% lower transcription levels of PcLRR-RK3 as compared to the wild type (LT263) (Figure 2a).

The vegetative development was characterised in Petri dishes to identify whether silenced transformants (T5 and T10) influenced the growth of *P. capsici*. The mycelium growth was documented by measuring the diameter of the colony up to 3 days of incubation. The digital photos and data were recorded after 24 h. The relative mycelial growth of two transformants is presented in (Figure 2b,c). The total colony diameter of T5 was significantly smaller than WT and there was a trend towards lower diameter at all-time points studied. Similarly, the colony diameter in T10 was also smaller than WT, but it was a little higher than the T5. This can be correlated with the difference in transcriptional expressional level of PcLRR-RK3 in both transformants. Taken together, these results indicated that vegetative mycelium growth was affected by PcLRR-RK3-silencing but to a different extent based on the gene expression level.

### 3.2. Impact of PcLRR-RK3 on zoosporangium development and production

**Figure 3.**
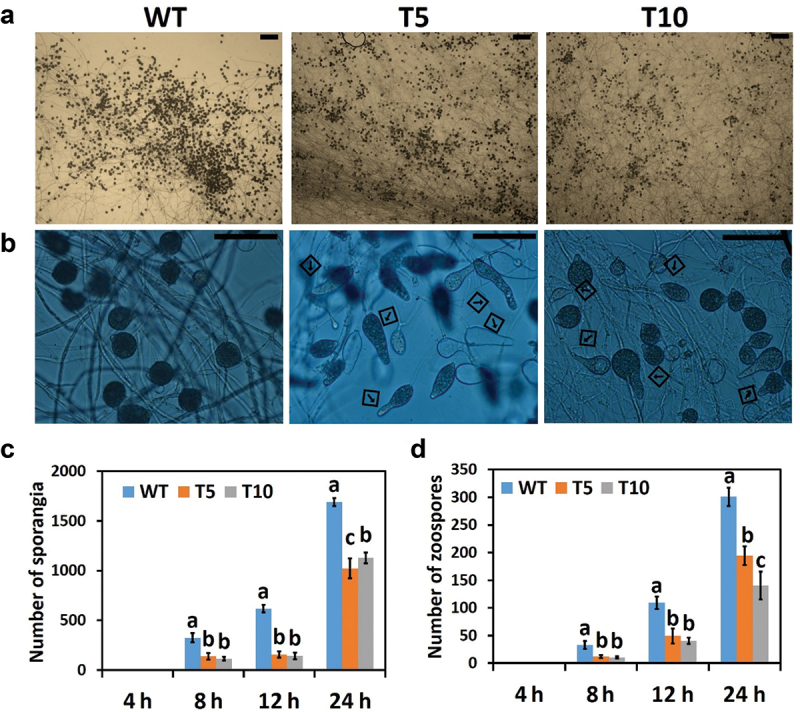
The vegetative growth was affected by PcLRR-RK3-silencing in *Phytophthora capsici*. (a) The image shows reduced number of sporangia by *PcLRR-RK3-*silenced transformants (T5 and T10); scale bars: 20 μm. (b) The image showing abnormal morphology of sporangia by *PcLRR-RK3-*silenced transformants (T5 and T10); scale bars: 20 μm. (c) Sporangia number was decreased by the PcLRR-RK3-silencing (T5 and T10 transformants) recorded at 4, 8, 12, and 24 h intervals. (d) Zoospores number was decreased in T5 and T10 transformants counted after 4, 8, 12, and 24 h intervals.

To study the effect of PcLRR-RK3 on asexual development, DIC images were taken at four time points following the incubation of silenced transformants (T5 and T10) and wild type. The overall zoosporangium morphology including sporangium formation and zoospore production from 4 h to 24 h has shown in Figure 3. We observed that two silenced transformants (T5 and T10) started producing sporangium at 8-h similar to wild type (WT) but the number of sporangia was significantly lower in silenced transformants (Figure 3a,c). Sporangium number kept on increasing with the time (12 h and 24 h) in all three strains studied (WT, T5, and T10) as a normal growth pattern, but the trend of the lower number of sporangia in transformants than in WT was sustained. The maximum number of sporangia was obtained at 24 h of incubation, which showed that differences in time also influence the growth rate of the pathogen. The microscopic studies revealed that silenced transformants lack the typical morphology of sporangia which are elongated at the apex and deformed as compared to the WT strain (Figure 3b). Whereas, the wild-type sporangia retained a typical ovoid to fusiform shape with a single papilla at the terminus (Hardham and Hyde 1997).

Sporangia are asexual means of reproduction in oomycetes by either germinating directly or differentiating into uninucleate zoospores after cytoplasmic cleavage. To check sporangium germination, we also quantified the number of zoospores produced by these strains (WT, T5, and T10). The number of zoospores was significantly lower in silenced transformants at all time points (4, 8, 12, and 24 h) as compared to wild-type strain (Figure 3d). This can be correlated with the fact that abnormal sporangia led to impaired cytoplasmic cleavage with abnormal zoospore production (Walker et al. 2008). These results simultaneously indicated that all stages of *P. capsici* growth were affected by silencing of *PcLRR-RK3.*

### 3.3. *PcLRR-RK3 is required for full virulence and in planta growth of* P. capsici

**Figure 4.**
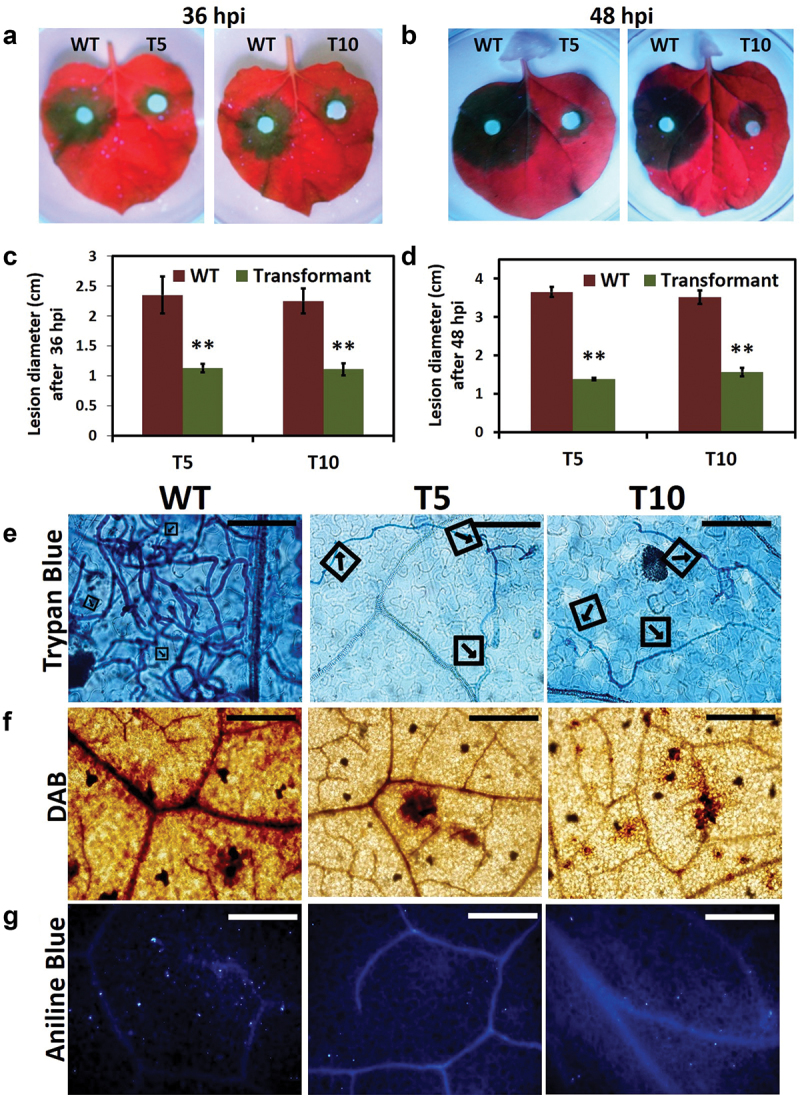
PcLRR-RK3is required for full virulence and *in planta* growth of *Phytophthora capsici. In planta* growth of WT and transformants at 36 hpi (a) and 48 hpi (b) under UV light. Size of lesions produced after transformants and WT inoculation at 36 hpi (c) and 48 hpi (d). Data was compared using Student’s *t*-test; ** indicated *P* < 0.01. (e) Trypan blue staining of the inoculated *Nicotiana benthamiana* leaves was done at 24 hpi. The typical photographs were taken after decolorizing with chloral hydrate; scale Bar = 30 µm. (f) DAB staining of the *P. capsici* inoculated *N. benthamiana* leaves was done at 12 hpi. Leaflets of 20-day-old *N. benthamiana* plant were inoculated with mycelial plugs from WT and silenced transformants (T5 and T10) at 25 °C. Photographs were taken after de-colorization of leaves with ethanol. The experiment was repeated three times with similar results. Three leaves were used for each treatment in each experiment. (g) Callose deposition was detected with aniline blue staining. Leaflets of 20-day-old *A. thaliana* plants are inoculated with mycelial plugs of WT and silenced transformants (T5 and T10) at 25 °C. Leaves were stained with aniline blue at 12 hpi. Ten biological replicates were used. Scale bars = 30 µm.

The virulence assay was performed using hyphal plug inoculums of all strains (WT, T5, and T10) on *N. benthamiana* leaves. We found that silenced transformants (T5 and T10) inoculated leaves showed significantly reduced disease lesion development at both time points studied 36 hpi (Figure 4a,c) and 48 hpi (Figure 4b,d), wild-type strain showed typical necrotic lesion which kept on increasing in diameter with time. These results showed that *P. capsici* virulence was also reduced with silencing of *PcLRR-RK3*.

To understand the mechanism behind the reduced virulence of silenced transformants, we performed trypan blue staining, H_2_O_2_ accumulation, and callose deposition assays in infected leaves to visualise the penetration, invasion, and infection of hyphae. In trypan blue staining, abundant hyphae growth was observed in leaf tissues infected with wild type (WT). However, very few hyphae were observed in the leaf tissues infected with PcLRR-RK3-silenced transformants (Figure 4e). Similarly, very weak DAB staining was observed in leaves inoculated with silenced transformants, whereas dark staining was observed in leaf tissues inoculated with wild type (WT). These results showed that H_2_O_2_ accumulation in PcLRR-RK3-silenced transformants (T5 and T10) was significantly lower than that in the wild type (WT). The mild staining of silenced transformants with DAB indicated that PcLRR-RK3-silenced transformants had failed to manipulate H_2_O_2_ production) (Figure 4f).

In the callose deposition assay, very few deposits were observed in leaf tissues inoculated with PcLRR-RK3-silenced transformants (T5 and T10). Substantially, leaf tissues inoculated with infectious hyphae of wild type (WT) showed more strong signals of callose deposits (Figure 4g). These results showed that hyphal growth of PcLRR-RK3-silenced transformants was significantly slower and weaker, which could not trigger the host immune response which was demonstrated by DAB and aniline blue staining. Taken together, all these results indicated that PcLRR-RK3 plays an important role in virulence by affecting host penetration and invasion of *P. capsici* mycelium as one of the earliest plant immunity responses to biotic stress is oxidative burst, callose deposition, and expression of defence-related genes (Boller and Felix 2009). We hypothesised that reduced virulence was due to the plant immune response, but these results showed that PcLRR-RK3-silenced transformants (T5 and T10) did not influence the host defence responses.

### 3.4. PcLRR-RK3 is essential for zoospore’s penetration and establishment into host leaf tissues

**Figure 5.**
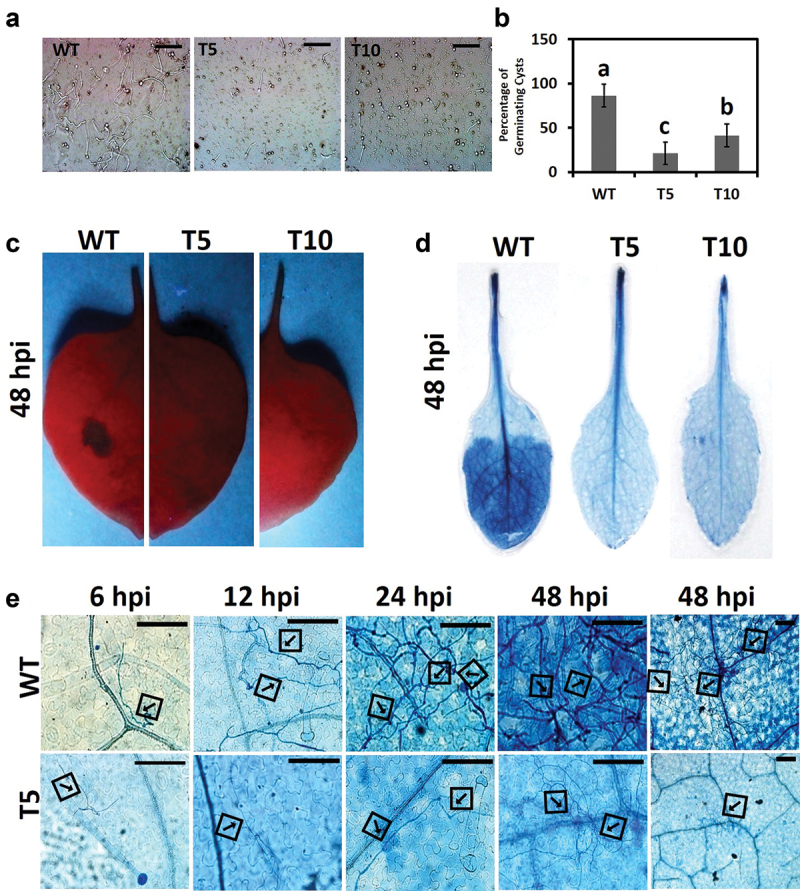
*PcLRR-RK3* is essential for zoospore penetration and establishment into host leaf tissues. (a) Micrographs showing the germinating cysts in the three strains (WT, T5 and T10). Scale bar = 30 μm. (b) The percentage of germinating cysts is reduced in silenced transformants. Scale bar = 30 μm. Pathogenicity assay was performed using the zoospore suspension. *PcLRR-RK3*-silenced transformants (T5 and T10) failed to form necrotic lesions in tobacco leaves (c) and Arabidopsis leaves (d). Leaves were inoculated with 10 μL suspension containing 100 zoospores/μL. (e) Micrographs showed the cytological zoospores development into host tissues (*Nicotiana benthamiana*). Germinating zoospores of silenced transformants could not form invasive hyphae to enter into host tissues. Leaves were spotted with 10 μL zoospores suspension (100 zoospores/μL) and zoospores development was fixed with trypan blue staining. The data were recorded at 6, 12, 24, and 48 hpi. Typical mycelium growth in host tissues is shown by arrows. Scale bar = 30 μm.

We examined whether *PcLRR-RK3-*silencing had affected the penetration and colonisation of *P. capsici* zoospores into host tissues. To test this hypothesis, we first tested the ability of zoospores to develop the germ tubes by placing the droplet of zoospore suspension in V8 broth *in vitro*. The wild-type strain started to form many long germ tubes after 2.5 h of incubation. In contrast, the zoospores of *PcLRR-RK3-*silenced transformants (T5 and T10) germinated poorly and developed fewer short germ tubes (Figure 5a,b).

To test the pathogenicity of zoospores, we inoculated host leaves with zoospores suspension of PcLRR-RK3-silenced transformants (T5 and T10) and wild type (WT). The results showed that silenced transformants did not produce lesions on the two host plants *N. benthamiana* (Figure 5c) and *A*. *thaliana* (Figure 5d) up to 48 hpi. We found that PcLRR-RK3-silenced transformants (T5 and T10) were non-pathogenic to both host plants while the wild-type strain was virulent and developed typical necrotic lesions on leaves.

To investigate whether loss of pathogenicity of zoospore inoculation was attributed to poor germination, we tested the formation of infection structures by PcLRR-RK3-silenced transformant (T5) and wild-type *P. capsici* (WT) on *N. benthamiana* leaves at different time points. At 6 hpi, wild-type zoospores started developing the germ tubes which kept elongated and penetrated host tissues later at 12 hpi. At 48 hpi, a high frequency of infectious hyphae formation and colonisation was observed in *P. capsici* (WT) (Figure 5e-upper panel). Whereas, PcLRR-RK3-silencing resulted in poor germination of zoospores with small and weak germ tube formation which failed to penetrate leaf tissues (Figure 5e-lower panel). These results indicated that the loss of pathogenicity of PcLRR-RK3-silenced transformants was caused by the defects in early zoospore germination stages. Collectively, we concluded that PcLRR-RK3-silencing weakens the ability of zoospore germination and penetration into host tissues, which is crucially required for the accomplishment of the infection process.

### 3.5. Sub-cellular localization of PcLRR-RK3

**Figure 6.**
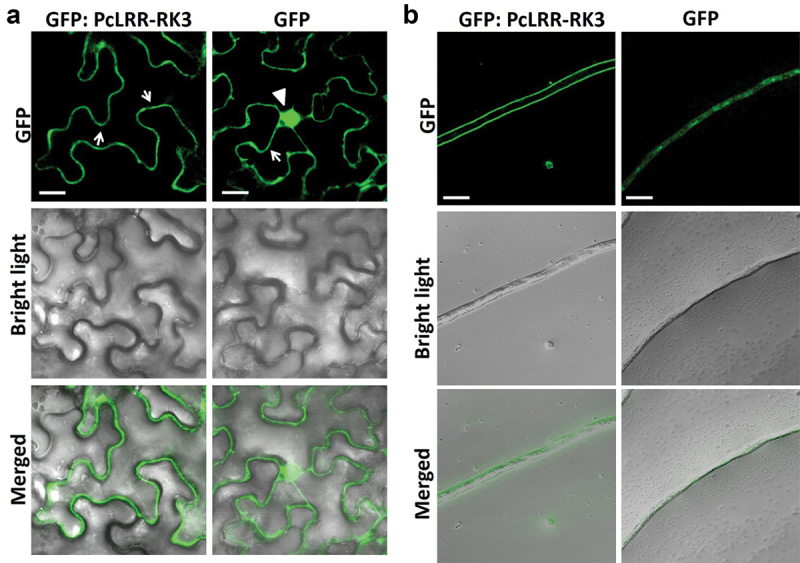
Sub-cellular localisation of GFP: PcLRR-RK3. (a) GFP fluorescence from GFP: PcLRR-RK3 fusion protein (left) and GFP alone (right) transiently expressed in *Nicotiana benthamiana* leaf epidermal cells using pBIN-GFP2 vector and its subcellular localization is shown. GFP fluorescence on plasma membrane and nucleus was indicated by arrow and arrowhead, respectively. Scale bar = 20 μm. (b) GFP fluorescence from GFP: PcLRR-RK3 fusion protein (left) and GFP alone (right) expressed into *P. capsici* mycelium. Scale bar = 20 μm.

As the predicted protein sequence of PcLRR-RK3 contained signal peptide, LRRs, transmembrane, and kinase domains, it was also supposed to be located in cell membrane like other LRR-RLKs investigated in plants (Osakabe et al. 2005; Germain et al. 2007; Jung et al. 2015). We transiently expressed the GFP-tagged PcLRR-RK3 in *N. benthamiana* leaves to verify its subcellular localisation using confocal microscopy. The transient expression of GFP: PcLRR-RK3 protein through *Agrobacterium*-infiltration of *N. benthamiana* leaves showed that GFP fluorescence was accumulated uniformly as a thin layer around the boundary layer of the cell (plasma membrane), whereas control GFP fluorescence was inconsistently detected in plasma membrane, nucleus, and cytoplasmic strands (Figure 6a). These results showed that PcLRR-RK3 was localised on the plasma membrane.

To better understand the subcellular localisation of PcLRR-RK3, the C-terminal sequence of PcLRR-RK3 was fused with the GFP gene and transformed into *P. capsici* cells by PEG-mediated transformation method. We used confocal microscopy to examine the positive overexpressed transformants fused with GFP: PcLRR-RK3. *P. capsici* transformed with GFP alone was used as a control. Results showed that green fluorescence of GFP: PcLRR-RK3 was localised to the plasma membrane only and absent in the cytosol and nucleus (Figure 6b). While the green fluorescence in the control (GFP alone) revealed that GFP was accumulated to cytosol and nucleus (Figure 6b). Based on these results, we concluded that the PcLRR-RK3 protein might play a potential role in the plasma membrane of cells as shown by others (Wu et al. 2015).

## 4. Discussion

LRR-RLKs being a large class of proteins perform various biological functions in the growth and development of plants (Torii et al. 1996; Lease et al. 2001). Generally, the mechanism of action is common in all LRR-RLKs, where LLRs domains provide ligand-binding sites and initiate signal propagation through the transmembrane domain on the membrane and induce conformational changes in the kinase domain, which starts the interaction with other RLKs forming homo- or heterodimers. Dimerised RLKs then undergo phosphorylation with the activation of the kinase and provide the docking sites for downstream phosphorylation targets (Chinchilla et al. 2007; Schulze et al. 2010; Roux et al. 2011).

Since, PcLRR-RK3-silenced transformants showed reduced pathogenicity which is possibly owing to the impaired growth of transformants. By trypan blue staining, we found that hyphae could not extend within host tissues and failed to generate the plant immune responses (oxidative burst and callose deposition). LePRK2, a pollen receptor kinase is located in the plasma membrane of tomato (Kim et al. 2002). During the start of pollen germination, the leucine-rich repeats portion of LePRK2 provides the interacting site to the extracellular protein (LAT52) of pollen (Tang et al. 2002). LePRK2 also regulates pollen tube growth by participating in the transduction of responses to extracellular growth-promoting signals. LePRK2 also impaired the Ca^2+^ responses which are necessarily required to maintain the polarity of the pollen tube during germination (Zhang et al. 2008). In another study, AtPRK2, a receptor-like protein kinase in complex with Rho guanine nucleotide exchange factors (RopGEFs) positively regulates the ROP1 signalling pathways by activating RopGEF1 through phosphorylation and controlling the pollen tube growth in *Arabidopsis* (Chang et al. 2013). Pollen tubes are plant sperm-carrying tubules during sexual reproduction that transfer the sperm cells to the embryo sac for fertilisation and multiplication of the organism. Similarly, a germ tube in *P. capsici* is also a means to enter and multiply within the host tissues. In our studies, we found that PcLRR-RK3-silencing resulted in poor germination of zoospores with small and weak germ tube formation which failed to penetrate leaf tissues. These results indicated that the loss of pathogenicity of the PcLRR-RK3-silenced transformants was caused by the defects in early zoospore germination stages. We also found that PcLRR-RK3-silencing weakens the ability of zoospore germination and penetration into host tissues, which is crucially required for the accomplishment of the infection process. These results suggested a possible relationship between PcLRR-RK3 and zoosporogenesis, which is further confirmed by Si et al. (2021) explaining that LLR-RLKs in *Phytophthora sojae* are directly affecting the zoospores chemotaxis and production.

The developmental stages and virulence in *Phytophthora* spp., are regulated by the expression and interaction of a large set of genes (Feng et al. 2010, 2014; Li et al. 2013, 2014). Many studies have been performed to explain the significant role of different proteins in the development and biology of *Phytophthora* spp. such as Phospholipase D (PLD) which was reported to generate the phosphatidic acid that induces the zoospore encystment (Latijnhouwers et al. 2003). Annexin which abundantly existed in the mycelium of *Phytophthora* species and encoded a typical type-2 calcium-binding motif [GxGT-(38 residues)-E] (Benz and Hofmann 1997) and involved in the pathogen adhesion to the host during infection (Meijer et al. 2006). Similarly, cellulose binding elicitor lectin (CBEL) functioned in adherence of the pathogen with the host as did the cellulose binding and lectin-like activity in *P. parasitica* (Gaulin et al. 2002). In another study, 31 proteins were found associated with germinating cysts, appressoria, and mycelium of *P. infestans* which includes the members of the CBEL family, the elicitin family, the Crinkler (CRN) family, and two transglutaminases, small Rab-type G-proteins, mucins, cell wall-associated enzymes and annexin (Grenville et al. 2010). One possible explanation would be that *PcLRR-RK3*-silencing has significantly affected the differential expression pattern of *P. capsici* developmental genes resulting in decreased and abnormal growth patterns.

In another study, LRR-RLKs in plants worked in complex with G proteins by dynamic interactions with FLS2/BAK1 and AtRGS1 resulting in changes in reactive oxygen species and calcium (Ca^2+^) release (Ozdemir et al. 2017). G proteins also coordinate with Ca^2+^ signalling pathways in oomycetes (*P. sojae*) during zoospore germination (Hua et al. 2008). Although plants and oomycetes belong to different kingdoms, we can hypothesise that the working model of LRR-RLKs could share some common features in both classes as LRR-RLKs possessed similar protein sequence features.

Consistent with LRR-RLKs in plants, PcLRR-RK3 was also located on the plasma membrane (Osakabe et al. 2005; Jung et al. 2015). Usually, many receptor-like kinases (RLKs) are reported to localise at the cell surface as these are associated with cell-to-cell interactions (Shiu and Bleecker 2001). Receptor-like kinases (RLKs) mediated signalling starts with induction of cellular differentiation with activation of various specific pathways leading to regulation of several biological processes involved in maintaining the shape of plants (Smet et al. 2009). Based on these interpretations, we can also propose that the PcLRR-RK3 protein might play a potential role in signalling mechanisms at the plasma membrane of the cell. Overall, our studies provided the insight that LRR-RLKs are also needed to explore in oomycetes, how they express and interact with other ligands at cell membrane/surface during the development and growth of organisms as studied in plants (Liao et al. 2017).

## 5. Conclusions

In this work, we have selected PcLRR-RK3 and characterised its functional importance in *Phytophthora capsici. PcLRR-RK3-*silenced transformants showed impaired morphology and virulence of *P. capsici*. The gene silencing also weakens the ability of zoospore germination and penetration into host tissues and fails to produce necrotic lesions. Altogether, we concluded that PcLRR-RK3 is required in the regulation of vegetative growth, zoospores penetration, and establishment into host leaf tissues.
